# Clinical effects of Cariprazine and their relationship with polymorphisms of dopamine and serotonin receptors: preliminary results from a prospective study on schizophrenia and bipolar disorder

**DOI:** 10.1192/j.eurpsy.2022.1746

**Published:** 2022-09-01

**Authors:** M. De Pieri, E. Dyrmishi, E. Bolla, M. Preve, R.A. Colombo, R. Traber

**Affiliations:** Organizzazione socio-psichiatrica cantonale, Psychiatry, Mendrisio, Switzerland

**Keywords:** real world setting, Antipsychotics, Precision Medicine, pharmacogenomics

## Abstract

**Introduction:**

Cariprazine (CAR) is a D2, D3, 5HT1A receptor partial agonist and a 5HT2A, 5HT2B antagonist, used to treat Schizophrenia and Bipolar disorder. Interindividual variability in therapeutic and side effects of antipsychotics is difficult to predict, due to non-genetic and genetic factors. Single nucleotide polymorphisms (SNPs) are the main source of genetic variability, the ones in dopamine and serotonin receptors to which CAR binds are indeed likely to determine response to treatment.

**Objectives:**

The aim of the study is to define a relationship between CAR clinical efficacy and SNPs in dopamine and serotonin receptors genes of patients affected by schizophrenia and bipolar disorder.

**Methods:**

We recruited 16 patients starting a monotherapy with CAR, evaluated at baseline and after 2, 4 and 8 weeks through BPRS rating scale. We selected a panel of SNPs in DR2, DR3, 5HT1A and 5HT2A receptors, with a frequency higher that 10% in Caucasians and functionally characterized. Cut-off for response to treatment was a 50% reduction of BPRS score. Statistical analysis was performed with one-way ANOVA followed by the test for linear trend between columns.

**Results:**

All subjects achieved response after 8 weeks of treatment, but 6 patients after 4 weeks. Early responders have a genetic profile associated with increased dopamine and serotonin receptor expression and/or binding affinity for their specific ligands. The association don’t reach statistical significance, probably due to low number of patients.

**Conclusions:**

Preliminary results suggest that an array of dopamine and serotonin receptors SNPs could predict time to respond to CAR in schizophrenia and bipolar disorder.

**Disclosure:**

The study is founded by Recordati AG, that commercialize the drug under study (Cariprazine) in Switzerland. Funding covers the costs for genetic analysis and other procedures of the study, no financial compensation is planned for investigators/authors.

